# Meta-analysis of Pandemic *Escherichia coli* ST131 Plasmidome Proves Restricted Plasmid-clade Associations

**DOI:** 10.1038/s41598-019-56763-7

**Published:** 2020-01-08

**Authors:** Kira Kondratyeva, Mali Salmon-Divon, Shiri Navon-Venezia

**Affiliations:** 1 Department of Molecular Biology, Faculty of Natural Sciences, Ariel University, Ariel, Israel; 2 The Dr. Miriam and Sheldon G. Adelson School of Medicine, Ariel University, Ariel, Israel

**Keywords:** Computational biology and bioinformatics, Microbiology

## Abstract

Extraintestinal multidrug resistant *Escherichia coli* sequence type (ST) 131 is a worldwide pandemic pathogen and a major cause of urinary tract and bloodstream infections. The role of this pandemic lineage in multidrug resistance plasmid dissemination is still scarce. We herein performed a meta-analysis on *E*. *coli* ST131 whole-genome sequence (WGS) databases to unravel ST131 plasmidome and specifically to decipher CTX-M encoding plasmids-clade associations. We mined 880 ST131 WGS data and proved that CTX-M-27-encoding IncF[F1:A2:B20] (Group1) plasmids are strictly found in clade C1, whereas CTX-M-15-encoding IncF[F2:A1:B-] (Group2) plasmids exist only in clade C2 suggesting strong plasmid-clade adaptations. Specific Col-like replicons (Col156, Col(MG828), and Col8282) were also found to be clade C1-associated. BLAST-based search revealed that Group1 and Group2 plasmids are narrow-host-range and restricted to *E*.*coli*. Among a collection of 20 newly sequenced Israeli ST131 CTX-M-encoding plasmids (2003–2016), Group1 and Group2 plasmids were dominant and associated with the expected clades. We found, for the first time in ST131, a CTX-M-15-encoding phage-like plasmid group (Group3) and followed its spread in the WGS data. This study offers a comprehensive way to decipher plasmid-bacterium associations and demonstrates that the CTX-M-encoding ST131 Group1 and Group2 plasmids are clade-restricted and presumably less transmissible, potentially contributing to ST131 clonal superiority.

## Introduction

Multidrug resistant ESBL-producing *Escherichia coli* ST131 is a high-risk extraintestinal pathogenic *E*. *coli* (ExPEC) lineage that has spread explosively throughout the world^1–4^, mostly by patient-to-patient spread and contaminated food^5^. This lineage may colonize healthy humans and animals^6^ and is the causative pathogen of urinary tract and bloodstream infections^7–9^. *E*. *coli* ST131 nowadays is recognized as the major *E*. *coli* lineage responsible for the spread of multidrug resistance (MDR)^3^ and in specific CTX-M ESBL genes^10^.

The global expansion of this lineage is driven by spread of two major fluoroquinolone-resistance clades which possess *fimH*30 allele - H30R/C1 and H30Rx/C2, where the later harbors *bla*_CTX-M-15_ ESBL gene^4,11–13^. Plasmid-bacterium associations in ST131 clades connected CTX-M-15-encoding IncF[F2:A1:B-] (FAB formula) plasmids to clade C2^11,12,14,15^, and non-CTX-M-15-encoding IncF[F1:A2:B20] plasmids to clade C1^4,11^. The major CTX-M genes described in clade С1 were CTX-M-14 and −27 alleles of the CTX-M-9 group^16^, with this later allele- C1-M27^16^, recognized recently as a new subclade with global occurrence^17,18^.

MDR often disseminates in bacteria through horizontal gene transfer of mobile genetic elements, especially plasmid transmission^19,20^. However, if there are highly adaptive plasmid-bacterium associations within ST131 lineage, the main driving force for MDR spread related to this pandemic clone is due to clonal expansion rather than to horizontal plasmid spread^11,12,15^. The existence of successful plasmid-ST131 lineage associations raises a question on the ability of this clone to serve as a reservoir for inter- and intra-species resistance spread. In spite several comprehensive studies on ST131 CTX-M-encoding plasmids, some questions are still understudied, including how frequently clade-associated plasmids are found in the respected clades? Are there plasmid horizontal transmission events between clades? What is the distribution of CTX-M genes across ST131 clades, and if there are correlations between specific clades, plasmid replicon types, and CTX-M gene alleles? Does ST131 serve as a MDR reservoir by transferring its most widespread plasmids to other bacterial species?

To address these questions, we explored ST131 plasmidome by performing a meta-analysis encompassing all ST131 whole genome sequencing data (WGS, n = 880) from the NCBI Sequence Read Archive (SRA). We assessed correlations between ST131 clades, plasmids types and CTX-M gene alleles. We focused on the high prevalent Group1 and Group2 plasmids, Group1 are clade C1-associated-CTX-M-27 IncF[F1:A2:B20] plasmids, and Group2 are clade C2-associated-CTX-M-15-encoding IncF[F2:A1:B-] plasmids.

In addition, we sequenced a collection of Israeli ST131 plasmids (n = 20) encoding CTX-M-15, CTX-M-14 and CTX-M-27 alleles, studied their diversity and verified plasmid-clade associations observed in the global dataset. We found a new CTX-M-15 phage-like plasmid group and screened the SRA collection for it. This study reveals insights on *E. coli* ST131 plasmidome; it improves our understanding of plasmid-associated traits within this lineage and sheds light on the ability of this pandemic clone to serve as a source for MDR plasmids.

## Results

### *E. coli* ST131 plasmidome

**Figure 1:**
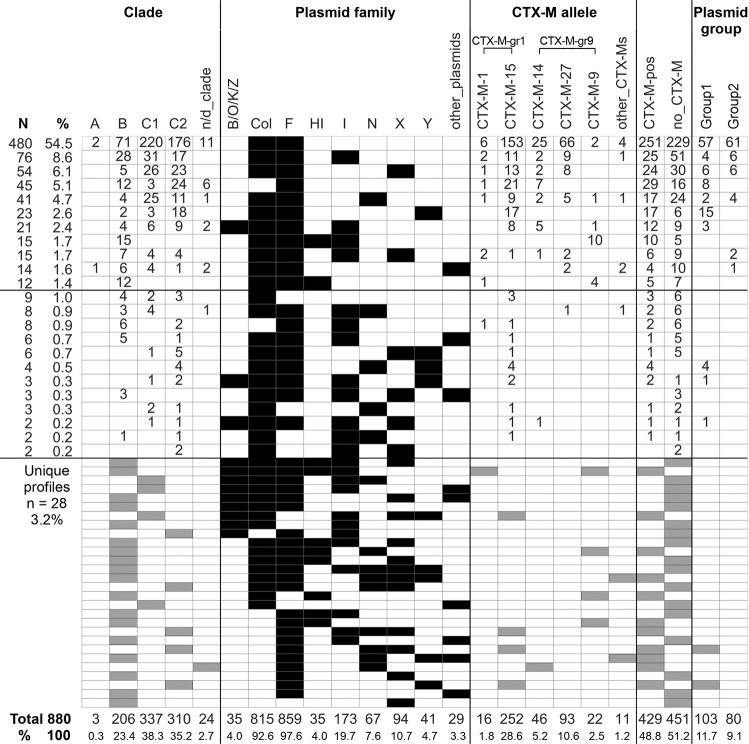
Plasmidome-based characterization of SRA ST131 isolates. ST131 isolates (SRA, n = 880) were divided according to the presence of each plasmid family (black). For each plasmidome profile, the distribution of clades, the presence of CTX-M genes, CTX-M alleles, and the presence of Group1 and Group2 plasmids is shown. Numbers represent the number of isolates with the respective properties. Single isolates possessing unique plasmid profiles are presented in Grey color. The total number of isolates are presented in the bottom of the Table in numbers and percent (out of 880 isolates). Plasmid families and CTX-M alleles found in less than 10 isolates are combined and presented as ‘other_plasmids’ and ‘other_CTX-Ms’, respectively. n/d_clade – clade was not identified; other_plasmids: A/C, L/M, P6, Q1, R, Rep, p0111, pEC4115, pXuzhou21; other_CTX-Ms: CTX-M-3, −32, −52, −55 (CTX-M-gr1); CTX-M-2 (CTX-M-gr2), CTX-M-19 (CTX-M-gr9); CTX-M-pos – CTX-M gene positive; no_CTX-M – CTX-M gene negative.

In order to explore plasmid-bacterium associations in *E*. *coli* ST131 lineage, we retrieved ST131 WGS data from the NCBI Sequence Read Archive (SRA, n = 880). We analyzed *in silico* each ST131 isolate for various bacterial host and plasmid features including the clade type, *fimH* and CTX-M alleles, plasmids’ replicon types and specifically IncF replicons of Group1 (IncFII_1, IncFIA_2, IncFIB_20) and Group2 plasmids (IncFII_2, IncFIA_1). The plasmidome of ST131 was highly diverse and revealed multiple combinations of plasmid types, which we present on a plasmid family-basis, i.e. combining IncFIA, IncFIB, IncFIC, and IncFII specific replicons and all their type into the IncF family (Fig. 1). About half of the isolates (49%) harbored CTX-M-encoding plasmids. The majority (90%) of all ST131 isolates carried Col-like and IncF plasmids either alone or co-carried with other plasmid families (I, N, X, Y or other), representing 93.2% of all CTX-M-encoding isolates. With respect to the CTX-M alleles, we found a highly conserved reservoir of CTX-M genes encoded in ST131, with only five major alleles belonging to the CTX-M-1 and CTX-M-9 groups (Fig. 1).

All SRA ST131 isolates that we positively filtered as possibly harboring Group1 plasmids, strictly belonged to clade C1 *fimH*30 (n = 80), whereas Group2 plasmid carriers were from clade C2 (n = 103), almost all possessing *fimH*30 (except for four isolates, two carrying *fimH*35 and two *fimH*-negative). For simplicity, possible Group1 and Group2 plasmid carriers will be further referred as ‘SRA Group1’ and ‘SRA Group2’ isolates, respectively.

Searching for Group1 and Group2 replicons only independent with the CTX-M alleles revealed their presence in 245 and in 164 SRA isolates, respectively (Supplementary Table S1).

### Associations between characteristics of *E*. *coli* ST131 and its plasmidome

To support plasmid-clade associations and to infer correlations between clades, plasmid types and CTX-M alleles in ST131 lineage, we estimated the strength of association between each pair of features using phi coefficient (Supplementary Fig. S1). Table 1 shows the results of the statistical analysis of these associations in clades C1 and C2. Indeed, Group1 and Group2 plasmid markers were tightly connected to specific clades, but also there were peculiar associations between clade C1 and plasmids Col156 (which was negatively correlated with clade C2), Col8282, and Col(MG828). These Col-like plasmids were highly abundant among ST131 SRA isolates, suggesting their potential importance in shaping the evolution of ST131 lineage and specifically clade C1. Principal-component analysis also showed that Group1 and Group2 SRA isolates were clustered separately, each possessing distinct combinations of features (Supplementary Fig. S2).

**Table 1:** Correlations between clades, plasmid types and CTX-M genes in ST131 SRA database (n = 880).

Clade	Category	Feature	N	phi coefficient	Corrected P-value*
C1 (n = 337)	Plasmid types	Col(MG828)	530	0.15	0.015
		Col156	524	0.52	<0.001
		Col8282	307	0.27	<0.001
		FIA	666	0.37	<0.001
		FIB	661	0.2	<0.001
		FIC	578	0.21	<0.001
		HI2	34	−0.16	0.0038
		HI2A	32	−0.15	0.0077
	IncF replicons	FIA_2	347	0.85	<0.001
		FIB_20	323	0.78	<0.001
		FII_1	327	0.71	<0.001
		FIA_1	241	−0.42	<0.001
		FII_2	272	−0.31	<0.001
		no_IncF	202	−0.39	<0.001
	CTX-M	CTX-M-27	93	0.43	<0.001
		no_CTX-M	451	0.17	0.0013
		CTX-M-15	252	−0.47	<0.001
	Grouped plasmids	Group1	80	0.4	<0.001
		Group2	103	−0.29	<0.001
C2 (n = 310)	Plasmid types	FIA	666	0.26	<0.001
		Y	41	0.18	<0.001
		Col156	524	−0.24	<0.001
		FIB	661	−0.44	<0.001
		HI2	34	−0.15	0.016
		HI2A	32	−0.14	0.03
	IncF replicons	FIA_1	241	0.61	<0.001
		FII_2	272	0.36	<0.001
		FIA_2	347	−0.52	<0.001
		FIB_20	323	−0.43	<0.001
		FII_1	327	−0.4	<0.001
	CTX-M	CTX-M-15	252	0.82	<0.001
		CTX-M-27	93	−0.25	<0.001
		no_CTX-M	451	−0.46	<0.001
	Grouped plasmids	Group2	103	0.49	<0.001
		Group1	80	−0.23	<0.001

*P-value of phi coefficient estimating the correlation between the presence of a clade and a plasmid feature after passing a P-value multiple test correction (step-down method using Bonferroni adjustments, alpha = 0.05)

### The complete sequences of Group1 and Group2 plasmids, and their identification in the SRA ST131 collection

**Figure 2:**
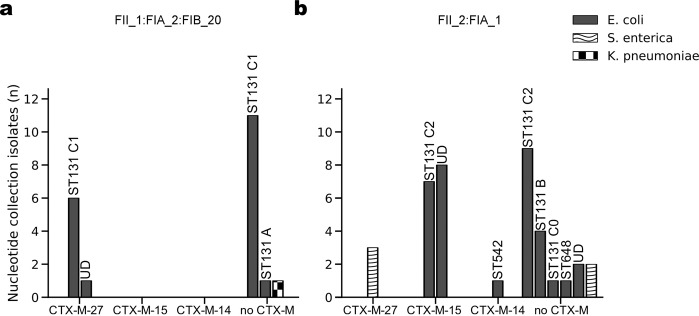
Distribution of Group1 and Group2 replicons in the Nucleotide collection. Bar graphs present the number of Enterobacteriaceae isolates carrying (**a**) Group1 plasmid replicons or (**b**) Group2 plasmid replicons mined from the Nucleotide collection (nt/nr). *E*. *coli* STs and clades are designated over the bars (when available). UD – undefined *E*. *coli* ST.

Mining the Nucleotide collection (nt/nr) using the target replicons and CTX-M genes returned 20 plasmids with Group1 replicon (IncFII_1, IncFIA_2, IncFIB_20, Fig. 2a) and 38 plasmids with Group2 replicon (IncFII_2, IncFIA_1, Fig. 2b). These two replicons mostly existed in *E*. *coli*, and although they were generally linked to the expected clades (C1 and C2), Group1 replicon could be rarely found in clade A and Group2 replicon in Clade B as already been observed in the SRA collection (Supplementary Table S1). Group1 (CTX-M-27, n = 7) and Group2 plasmids (CTX-M-15, n = 15) were found in *E*. *coli* only (Supplementary Table S2).

Interestingly, 5 out of the 7 Group1 plasmids co-encode Col156-replicon (154nt length). Co-existence of these two replicons on the same plasmid partially explains the high correlation rate of these two traits that we have observed in the SRA analysis (Table 1). The distribution of these replicons is overlapping, but there are ST131 isolates that carry plasmids encoding only one of the replicons, suggesting alternative routes of Col-replicon spread.

Furthermore, we wanted to select Group1 and Group2 complete sequence reference plasmids and to use them for SRA WGS read mapping in order to examine if ST131 SRA isolates contain these sequences. The following ST131 plasmids were chosen: Group1 – pEC-81009 (135.7 Kb, CP021180) from clade C1, and Group2 - plasmid uk_P46212 (143.7 Kb, CP013657) from clade C2. BLAST-based comparison of plasmids from the NCBI Nucleotide database showed high similarity of sequences within groups and proved that the reference plasmids may serve as representative sequences for these groups: the average coverage of pEC-81009 by Group1 plasmids was 93.4%; and 95.7% for uk_P46212 covered by Group2 plasmids (Supplementary Fig. S3).

Reads of Group1 SRA isolates (n = 80) covered Group1 reference plasmid to a higher breadth compared to Group2 reference (89.8 ± 9.5% vs. 53.7 ± 7.3%, P-value < 0.0001), and reads of Group2 plasmids (n = 103) to the Group2 reference (62.2 ± 6.7% vs 84.6 ± 8.7%, P-value < 0.0001; Supplementary Fig. S4), suggesting the presence of the homologous reference sequences within ST131 SRA data.

### Plasmid-clade associations among Israeli CTX-M-producing *E*. *coli* ST131 isolates

**Figure 3:**
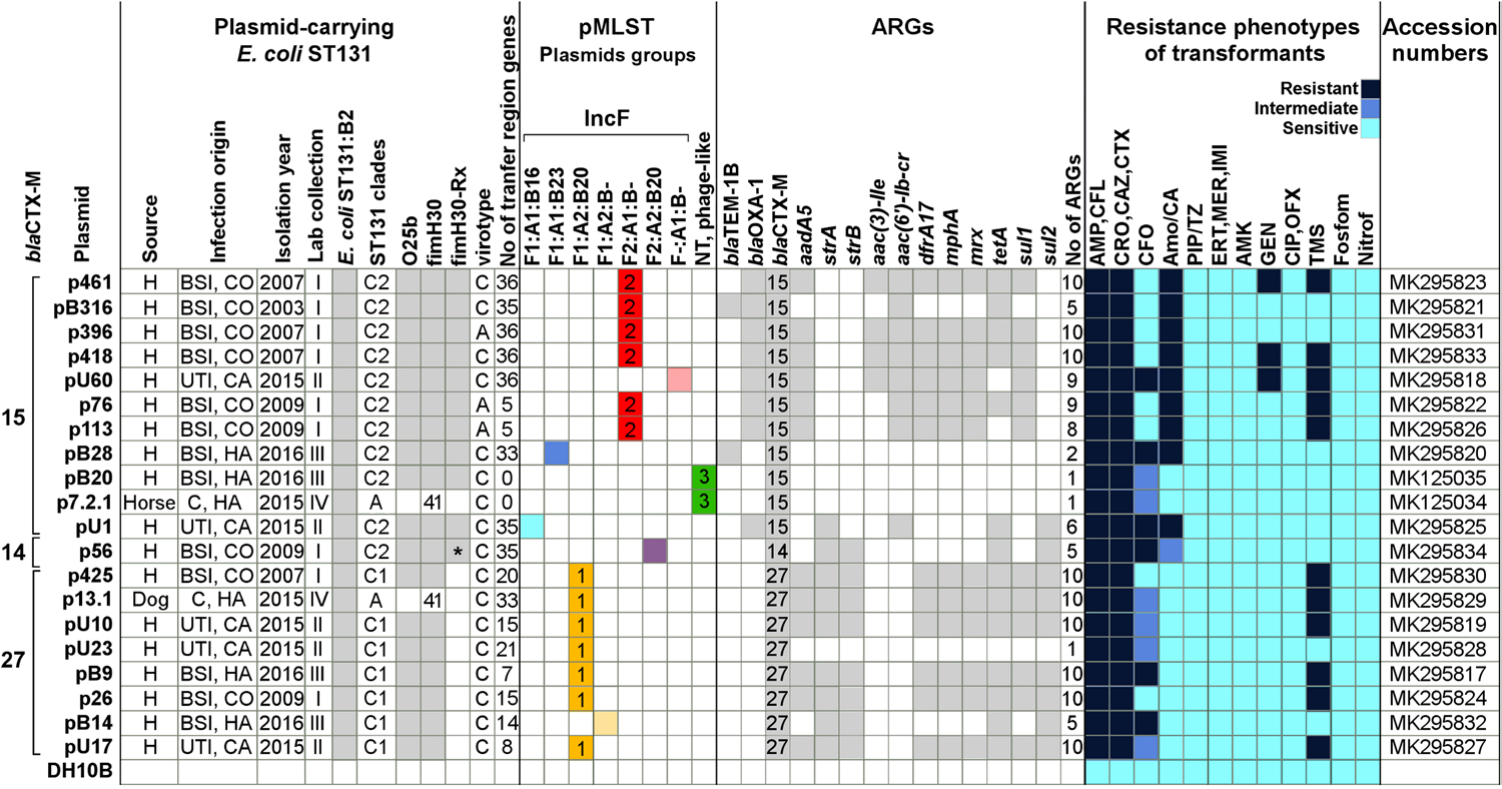
Characteristics of 20 sequenced *E*. *coli* ST131 CTX-M-encoding plasmids and traits of their ST131 carrying strains. Twenty CTX-M-encoding plasmids were purified from *E*. *coli* ST131 isolates recovered from different clinical sources and isolated from 2003 to 2016. These 20 isolates were selected from four different ESBL-producing strain collections in our lab (I to IV), and were subtyped by multiple PCRs. Plasmids replicon types and antibiotic resistance genes (ARGs) were determined *in silico*. Based on sequence similarities, plasmids were further grouped (designated 1 to 3 and marked by different colors). Purified plasmids were transformed into *E*. *coli* DH10B and resistance phenotypes determined by Vitek-2, are presented on the right. *E. *coli* ST131:H30Rx carrying p56 encoded two CTX-M genes, CTX-M-15 and CTX-M-14. Plasmid encoding CTX-M-14 was purified and sequenced. H, Human; BSI, blood stream infection; UTI, urinary tract infection; C, gut colonization; pMLST, plasmid multilocus sequence typing; NT, not typable; AMP - ampicillin, CFL - cefalexin, CRO - cefuroxime, CAZ - ceftazidime, CTX - ceftriaxone, CFO - cefoxitin, Amo/CA - amoxicillin/clavulanic acid, PIP/TZ - piperacillin/tazobactam, ERT - ertapenem, MER - meropenem, IMI - imipenem, AMK - amikacin, GEN - gentamicin, CIP - ciprofloxacin, OFX - ofloxacin, TMS - trimethoprim/Sulfamethoxazole, Fosfom - fosfomycin, Nitrof – nitrofurantoin.

*E*. *coli* ST131 arrived in Israel in 2003 causing mainly community-onset bloodstream infections^21^. To explore the local ST131 CTX-M-encoding plasmids and to compare them with the global ST131 plasmidome, we isolated CTX-M-encoding plasmids from 20 *E*. *coli* ST131 isolates from our collections (2003–2016, 18 clinical human isolates and two animal colonizing isolates, Fig. 3) and determined their complete sequences.

According to the specified replicon types, six CTX-M-15-encoding plasmids were classified as Group2, and seven CTX-M-27-carrying plasmids belonged to Group1. All grouped plasmids, except one, were isolated from *E*. *coli* ST131 hosts of expected clades. One peculiar exception was a subclone of clade A (*fimH*41) that colonized a dog and harbored Group1 plasmid (p13.1, Fig. 3). Col156-replicon was co-encoded by all, except one (pU17), Group1 plasmids.

The previously used reference plasmids were found to be highly similar to our newly sequenced grouped plasmids: pEC-81009 with all Group1 plasmids (>90% coverage and >99.8% maximum nucleotide identity) and uk_P46212 with Group2 plasmids (>92% coverage and >99.8% maximum nucleotide identity; Supplementary Fig. S5). Two of the Israeli sequenced plasmids lacking one of the target replicons (pB14 and pU60), although possessing homologous sequences (Supplementary Fig. S5), were not included in plasmid groups to focus only on plasmids with the specified genetic targets.

Out of the 20 sequenced plasmids, two nearly identical phage-like plasmids formed a new group, Group3. They shared 100% coverage and 99% identity with pECOH89 (HG530657; Supplementary Fig. S5) from an *E*. *coli* ST349 clinical isolate causing a wound infection in Germany in 2010 (personal communication). This is the first report on Group 3 plasmids in ST131 background, found in clades C2 and A. To determine the distribution of Group3 plasmids in ST131 population, ST131 CTX-M-15-encoding SRA isolates (n = 252) were mapped to Group3 reference, pECOH89. We found 18 isolates covering more than a half of the reference sequence, the coverage range was 83.2–94.3%. These isolates belonged to C1 (n = 3, two possibly co-carried with Group1 plasmids) and C2 (n = 15, four isolates possibly also harbored Group2 plasmids) clades.

Two out of the CTX-M-15-encoding plasmids (pU1 and pB28) and one CTX-M-14-encoding plasmid (p56), were novel and each belonged to a distinct plasmid group (Fig. 3).

### Plasmid annotation and general group features of ST131 Israeli collection

**Figure 4:**
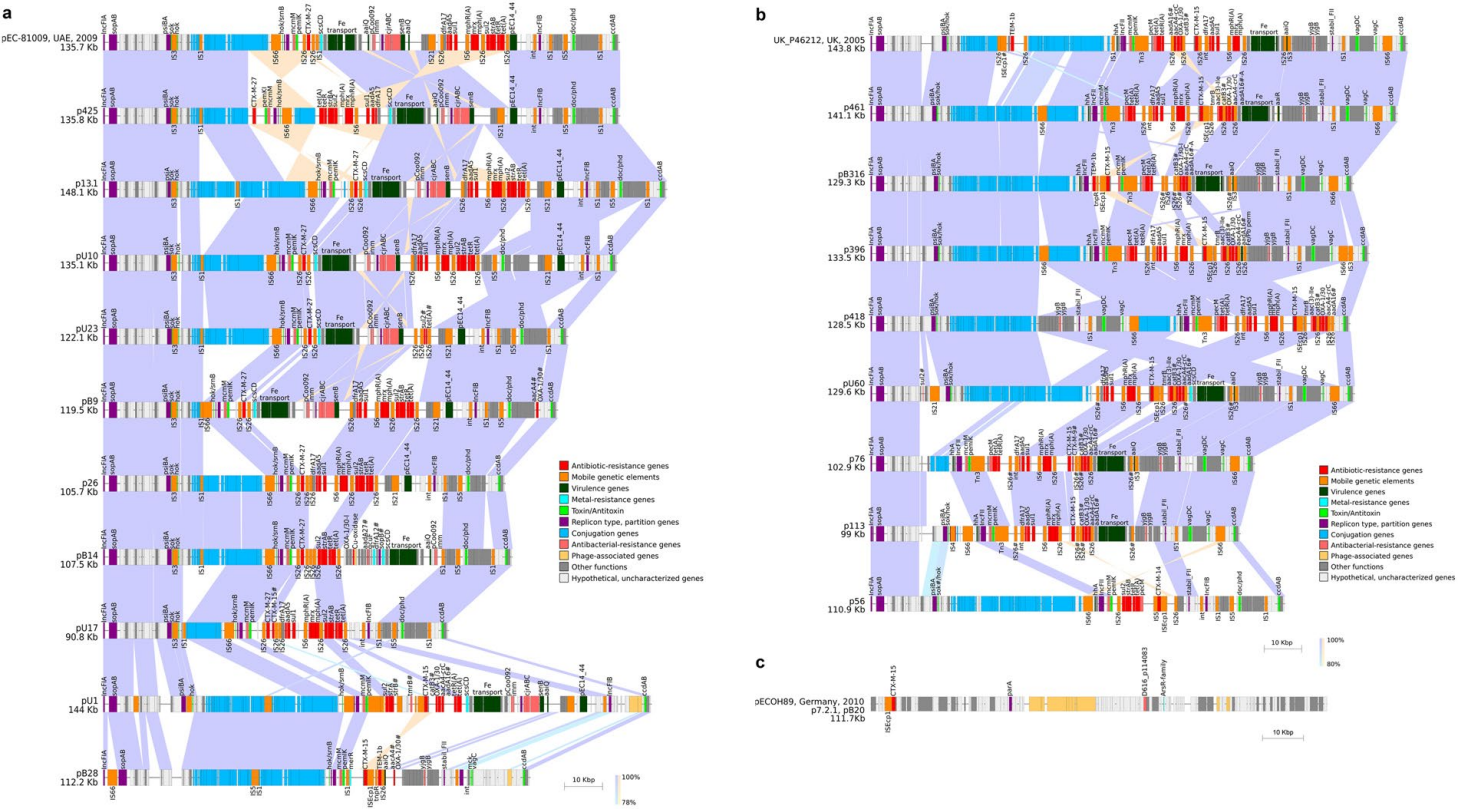
Linear maps of 20 *E*. *coli* ST131 CTX-M-encoding plasmids and highly related GenBank plasmids. Plasmids were grouped (Group1, **a**; Group2, **b**; or Group3, **c**) and the GenBank plasmid with the highest similarity (>95%) to each group member is presented at the top of each panel as the reference plasmid of this group. The three ungrouped plasmids (pB28, pU1 and p56) were included in panels **a** and **b** based on sequence similarity. A single scheme describes Group3 plasmids (n = 3) due to their high resemblance. Rectangles represent annotated genes (colored by function) and IS elements (recognized by ISFinder; IS26 and ISEcp1 are specified and other IS are family-based labeled). BLASTn-comparisons between neighboring plasmids were determined (purple, same direction alignment; orange, opposite direction). Scale bars are shown for each plasmid group.

All IncF plasmids (groups 1, 2 and ungrouped plasmids; n = 18) consisted of a plasmid backbone and an accessory region (Fig. 4). The major toxin-antitoxin systems in Group1 and Group2 plasmids were *ccdAB*, *pemIK* and *sok/hok* combined with *doc/phd* in Group1 plasmids and *vagCD* in Group2 plasmids. The Israeli plasmids varied significantly in the number of conjugation transfer genes (7–33 genes in Group1 and 5–36 genes in Group2, Fig. 3), with a reduction in the number of conjugation genes along the years.

In the accessory region, Group1 plasmids encoded antibiotic resistance genes (ARGs) cassettes (1–10 genes), and possessed diverse virulence-associated traits including an iron transport system “IncF island 1” described previously^11^, the colicin ColIa immunity-encoding genes *cjrABC*, the enterotoxin-encoding *senB* gene, the copper-sensitivity suppressor *scsCD*, and *merR*, the mercury resistance regulator. Group2 plasmids encoded ARGs (five to 10 genes) and the iron transport system mentioned above.

Group 3 plasmids consisted of phage-related and hypothetical proteins. These plasmids lacked conjugation region and stability systems. However, the appearance of this group in ST131 isolates of clades A and C suggests their possible horizontal transfer.

Hypothetical and uncharacterized proteins (i.e. not-present or uncharacterized genes in UniProtKB/Swiss-Prot database) encoded by our sequenced plasmids, comprise around 15–18% of length of IncF plasmids, and 40% of length of Group3 phage-like plasmids. These DNA regions are interesting to explore, and require the development of computational tools and molecular studies in order to predict their potential role, which may reveal their contribution to ST131 lineage.

### A phylogenetic analysis of grouped plasmids

**Figure 5:**
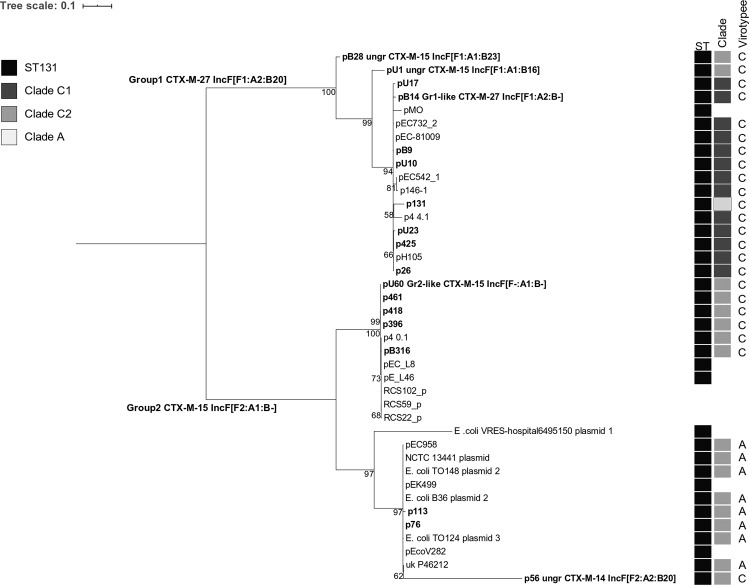
Phylogenetic relationships of IncF ST131 plasmids. Maximum-likelihood phylogenetic analysis of IncF plasmids (n = 40) based on the concatenated SNPs (n = 1368) from plasmid sequences. Unrooted tree was re-structured to show the divergence between Group1 (upper cluster) and Group2 (two lower clusters) plasmids. Israeli plasmids (n = 18, excluding Group3) are shown in bold. Ungrouped plasmids (‘ungr’) and Group1 or Group2-like plasmids are presented with their CTX-M alleles and replicon types. Bootstrap values (100 replicates) >50% are presented on the branches, where all branches with lower bootstrap values were deleted. If available, data on plasmid hosts was added showing host ST, clade and virotype. UD – *E*. *coli* ST is undefined.

The phylogenetic tree of grouped plasmids (Fig. 5) was based on IncF plasmid SNPs retrieved from the Nucleotide database and Israeli collection (n = 40). Group1 plasmids carried in ST131 clade C1 clinical isolates were evolutionary close, whereas p13.1 recovered from a ST131 clade A isolate colonizing an animal was distant. Group2 plasmids were separated into two branches; with Israeli plasmids present in both. ST131 clade C2 virotypes (A and C) clustered on different branches. Three Israeli plasmids with unique replicon types were added to the analysis. pB28 and pU1 were highly related to Group1 plasmids, whereas p56 appeared on the Group2 branches.

## Discussion

This study presents the first meta-analysis performed on SRA ST131 WGS data in order to explore ST131 plasmidome. We mined 880 SRA isolates to reveal correlations between plasmid and ST131 chromosomal features. We limited our analysis to several widespread CTX-M-carrying plasmid groups; however, the same approach could be applied to identify the distribution of any bacterial genetic markers.

ST131 plasmidome consisted mostly of IncF and Col-like plasmids, each presented in more than 90% of the studied SRA isolates. Less common plasmid families were found in 39% of the isolates, usually accompanied by the high-prevalent IncF and Col-like plasmids. One of our consistent findings was a clear segregation between specific IncF plasmid groups and ST131 clades. We found that: (i) Group1 plasmid features (CTX-M-27, FII_1, FIA_2, FIB_20) were restricted to ST131 clade C1 (with one exception in clade A); (ii) Group2 plasmid specific traits (CTX-M-15, FII_2, FIA_1) were restricted to clade C2 isolates exclusively. This study provides an extensive support to previously described combinations of ST131 clades and IncF plasmids^11,12,16^. IncF plasmids are characteristic for ST131, and the evolution of this lineage has been described in parallel to IncF plasmids arrivals and loss^4^.

The contribution of Groups 1 and 2 IncF plasmids on *E*. *coli* ST131 superiority have yet to be studied. In *E*. *coli* K12, a non-ST131 *E*. *coli* background, the acquisition of IncF Group2 plasmid resulted in minor chromosomal modifications and fitness cost diminished after several generations, while acquisition of IncC-IncR plasmid lead to major genomic rearrangements with consistent high fitness cost^22^. In *K*. *pneumoniae*, adaption to IncF pKpQIL-like plasmids was associated mostly with changes in gene expression levels, together with variations in other physiological features such as conjugation rates, competitiveness, or biofilm formation levels^23^. In another study that looked on the effect of CTX-M-15- and CTX-M-14-encoding plasmids on non-ESBL-producing ST131 strains, bacterial growth rates were not affected, whereas biofilm formation and serum resistance decreased after plasmid acquisition^24^. Unfortunately, the clades used in that study were not defined. To extend our understanding on plasmid-clade adaptation processes, comprehensive comparative studies on ST131 clades C1 and C2 transformed with Group1 and Group2 plasmids are required.

In contrast to the well-described IncF plasmids, the role of small Col-like plasmids in ST131 is still unknown in spite of their wide occurrence in this lineage. Examination on the most widespread Col replicons revealed correlations between Col156, Col(MG828), and Col8282 plasmids and clade C1, however all these mentioned replicons were found in both clades C1 and C2 co-existing with the strictly divided IncF Group1 and Group2 plasmids. Col156 plasmids were recognized previously as important ARGs transmitters^25,26^, and a wide distribution of Col-like plasmids was shown in the recently emerging clone *E*. *coli* ST1193^27^. However, further study is required to identify *E*. *coli* ST131 isolates carrying small Col156 plasmids, as well as to accurately distinguish them from isolates that co-encode Col-like replicons with other replicon groups. Less frequent plasmid families were identified in different ST131 clades. A major caveat of performing a meta-analysis that is based on Illumina WGS data is the problem of sorting chromosomal and plasmid reads. Thus, we were not able to divide SRA ST131 isolates into isolates that harbor multiple plasmids or one plasmid co-encoding fused replicons. This problem highlights the necessity of increasing the collection of available complete sequenced plasmids.

Data from this study expanded the global collection of CTX-M-encoding ST131 plasmids by 20 new complete sequences from diverse sources in Israel. Our clinical ST131 isolates belonged to clades C1 and C2 and harbored mostly Group1 and Group2 plasmids. However, we identified several plasmids with unique replicon types. Some of these plasmids showed high sequence homology to the grouped plasmids, like pB14 (IncF[F1:A2:B-]) and pU60 (IncF[F-:A1:B-]), possibly derivatives of Groups 1 and 2, respectively. A comparison of the genetic modifications and the host features of plasmids diverged from the globally widespread plasmid groups will improve our understanding on plasmid evolution, host range, and plasmid-host adaptation. Our CTX-M-encoding ST131 plasmid collection revealed a complicated epidemiology of ST131 in Israel. We found Group2 plasmids in clade C2 isolates; the phylogenetic analysis we performed divided Group2 plasmids into two subgroups in agreement with ST131 virotypes. In addition, we identified a new phage-like plasmid group (Group3), described herein for the first time in ST131. In our collection, Group3 plasmids were found in ST131 clades A and C2. This Group3 plasmid was originally described in *E*. *coli* ST349^28^, and in this meta-study, we found Group3 plasmids only in 18 ST131 SRA isolates carried in clades C1 and C2. These plasmids appear to be rare in SRA isolates compared to Group1 (n = 80) and Group2 (n = 103) SRA isolates, which highlights the clade-restricted nature of specific IncF plasmids.

In our newly sequenced plasmids, we noticed conjugation gene loss in both Group1 and Group2 plasmids, where the number of known conjugation genes dropped significantly (from 33 to seven genes with some intermediate numbers in Group1 plasmids and from 36 to five genes for Group2 plasmids). Conjugation genes loss was previously described in Group2 plasmid pEK958^29^. Based on the involvement of active conjugation machinery on bacterial fitness cost^30^, we speculate that plasmids that are associated with a specific ST131 clade that spread vertically may result in the reduction of *tra* genes. Nevertheless, the translational consequences of this reduction on fitness cost, plasmid transferability, and conjugation rates should be further elucidated experimentally.

Interestingly, we demonstrated that Group1 and Group2 plasmids may possibly be found in other ST131 clades as in the case of Group1 plasmid p13.1 isolated from ST131 clade A colonizing a dog. Our collection of ST131 clade A isolates was limited to only two samples, thus the plasmidome richness of rare ST131 clades is still unknown. Systematical studies have started to describe the epidemiology of ST131 from animal and environmental reservoirs^31,32^ however, further research should be performed to extend our knowledge.

In conclusion, we showed that ST131 plasmidome is shaped by clade-restricted plasmids. Available WGS databases serve as a challenging source to explore plasmid networks and spread patterns within *E*. *coli* ST131 or in other lineages and species. Although the entire ST131 plasmid network is not fully resolved, the combination of clinically important CTX-M-encoding MDR IncF plasmids within specific ST131 clades became conclusive. IncF plasmids could be ‘locked’ within their clades, however they still may exchange ARGs transmitted by IS elements^33^, which keeps ST131 as a threatening MDR reservoir.

## Methods

### Generation and *in silico* characterization of a global *E*. *coli* ST131 database

#### Generation of Group1 and Group2 complete sequence plasmid database

Complete sequence plasmids of Group1 and Group2 were retrieved from the NCBI Nucleotide collection (nr/nt). The concatenated target replicons and CTX-M genes (Group1 – IncFII_1, IncFIA_2, IncFIB_20 and CTX-M-27; and Group2 – IncFII_2, IncFIA_1 and CTX-M-15), separated by gaps of 50 Ns were submitted for the web BLASTn search (https://blast.ncbi.nlm.nih.gov/Blast.cgi). Then, sequences with full-length and 100% identical target regions were added to this study.

#### Generation of Sequence Read Archive (SRA) *E. coli* ST131 database

Whole genome sequences (WGS) from the NCBI Sequence Read Archive (SRA) were searched for “E. coli ST131”. *E*. *coli* genomes (n = 1336, last accessed at Dec 2018) were loaded excluding three samples that appeared also in the Nucleotide collection with identical BioSample numbers. The accurate identification of *E*. *coli* sequence types, ST131 clades, *fimH*-alleles, together with IncF replicon types, and the CTX-M alleles was performed using two complementing approaches: (I) reads were trimmed (Trimmomatic-0.36), assembled (SPAdes-v3.10.1) and then a complete target sequence was found by local BLASTn-based analysis, and/or (II) trimmed reads were mapped (bwa 0.7.17-r1188) to the reference genomes and analyzed using samtools-1.7 and bcftools-1.8, checking for 100% coverage and a total absence of SNPs in the area of interest. Samples were identified as ‘ST131’ if all seven Achtman MLST genes and *pabB* from Pasteur scheme were identical to ST131 alleles. Clade-specific regions were cropped from ST131 isolates - clade C0 CD306, clade C1 EC81009, clade C2 JJ1886 and EC958 (all coordinates are listed in Supplementary Table S3), or retrieved from the pre-assembled SRA isolates using clade A and B identification primers^34^. Databases of IncF replicons and CTX-M genes were downloaded from pMLST 2.0^35^ and ResFinder-2.1^36^ CGE Web tools.

Plasmid families were identified based on methods described above or by read mapping using KMA-1.1.7^37^ found ≥80% identity with a plasmid replicon from the CGE PlasmidFinder 2.0 Enterobacteriaceae collection^35^. Virotypes were identified based on the presence or absence of four genes included in the ST131 virotyping scheme^38^ and virulence genes were searched using BLASTn-based analysis and KMA-1.1.7. Reference plasmid coverage was identified by mapping SRA reads (bwa 0.7.17-r1188) followed by samtools depth analysis.

### Descriptive statistical analysis

In an attempt to find plasmid-bacterium associations, we constructed a binary table of ST131 isolates characteristics that describes the presence or absence of each studied allele. The association coefficient between each pair of ST131 characteristics was determined using phi coefficient (p-value < 0.05). *P*-values of coefficients were corrected in a *P*-value multiple test based on the step-down method using Bonferroni adjustments (alpha = 0.05). Principal-component analysis transformed characteristic binary data into contributing loading vectors placing SRA isolates on the scatter plot.

The samples of coverage results of grouped SRA isolates and the two reference plasmids were proved not to be normally distributed by the Shapiro-Wilk and Anderson-Darling tests; thus sample means were compared using the two-tailed Mann-Whitney test. *P-*values of < 0.001 were considered significant. All statistical analyses were performed using Python modules.

### *E*. *coli* ST131 bacterial strains and growth conditions

Twenty MDR CTX-M-producing *E*. *coli* ST131 isolated in Israel (2003–2016) were included in this study (Fig. 3). Isolates were selected from four different laboratory collections. Bacteria were grown on Luria-Bertrani (LB) agar plates or LB medium (HyLabs, Rehovot, Israel), supplemented with ampicillin (100 µg/mL) for maintenance of CTX-M-encoding plasmids.

### Molecular characterization of *E*. *coli* ST131 isolates

Selection of the 20 CTX-M-encoding *E*. *coli* ST131:B2 isolates was based on multiplex PCR methods including classification of the phylogenetic group^39^, identification of ST131^40^ and determination of CTX-M-group using multiplex PCR^41^ and sequencing (Syntezza, Israel). *E*. *coli* ST131 clades were determined by Matsumura’s scheme^34^. For clades C1 and C2, presence of O25b serotype^42^ and *fimH*30 allele^43^ were additionally verified. For non-*fimH*30 ST131 isolates, *fimH* gene was amplified and sequenced (Syntezza, Israel). A unique presence of *fimH*30:Rx SNP was also proved for *E*. *coli* ST131 clade C2^44^. Virotypes were identified by multiplex PCR^38^.

### Purification and transformation of ST131 CTX-M-encoding plasmids

CTX-M-encoding plasmids were purified from the 20 ST131 isolates using Plasmid Midi Kit (QIAGEN, Germany). Plasmids were individually transformed by electroporation (GenePulser, Bio-Rad, USA) into freshly prepared electro-competent *E*. *coli* DH10B, and resulted transformants were selected on LB plates supplemented with 100 μg/mL ampicillin. CTX-M-encoding plasmids were isolated from transformed *E*. *coli* DH10B and checked for the presence of accompanying plasmids from the clinical strains by gel-electrophoresis and plasmid incompatibility group typing^45^. To ascertain the purity of *bla*_CTX-M_-encoding plasmid for sequencing, additional transformations were carried out until purity was confirmed. The resulted CTX-M-encoding plasmids were cleaned from residual chromosomal DNA using Plasmid-Safe ATP-dependent DNase (Epicentre, USA). Antibiotic susceptibility testing of all 20 *E*. *coli* DH10B transformants carrying a single CTX-M-encoding plasmid was performed using Vitek-2 (bioMérieux, France).

### Plasmid sequencing, assembly and annotation

Plasmids were sequenced by Illumina HiSeq 2500 (Illumina, San Diego, CA, USA) at the Technion Genome Center (Technion Institute of Technology, Haifa, Israel) using a 2 × 250 bp paired-end libraries with an estimated fragment size of 800 bp. Adapter sequences and low-quality reads were removed using Trimmomatic-0.36^46^. Contamination of *E*. *coli* DH10B chromosomal DNA was filtered out using bowtie2–2.3.2 and samtools-1.7, if necessary. Where it improved assembly statistics, read coverage was normalized to 50x using BBMap/BBTools-36.20. Scaffolds were generated by SPAdes-3.10.1 assembling Illumina long paired reads^47^.

Four ST131 plasmids [p425 (Group1), p461 (Group2), pU1 and p56 (both ungrouped)] were additionally sequenced using Nanopore technology (Oxford Nanopore Technologies, ONT, Oxford, United Kingdom) to complete plasmid assembly^48^. Plasmids were sequenced using standard protocol SQK-RAD004 v. RSE_9046_v1_revB_17Nov2017 according to the manufacturer’s instructions. Libraries were prepared using the SQK-RAD004 ONT Rapid sequencing kit and loaded onto the MinION flow cell FLO-MIN106 (R9.4 SpotON). Adapter sequences were trimmed using Porechop (v0.2.3) and low quality reads were removed using NanoFilt (v.2.20). The hybrid read sets (both Illumina and Nanopore reads) were assembled using Unicycler (v0.4.0)^49^. SPAdes scaffolds of other plasmids were ordered using a home-made Python script according to the MinION sequenced reference or the GenBank highly related plasmid. Plasmid sequences were annotated with the RAST tool^50^. Predicted proteins were verified and updated using UniProtKB/Swiss-Prot database. ARGs and their transport cassettes were retrieved using ResFinder-2.1^36^, supported by CARD^51^ and ISfinder^52^. Virulence genes were identified using VirulenceFinder-1.2^53^ and VFDB^54^. Replicon types were identified using pMLST-1.4 and PlasmidFinder-1.2^35^. Plasmid maps were generated using Easyfig-2.2.3^55^.

### Nucleotide sequence genbank accession numbers

The complete sequences of 20 plasmids have been deposited at the GenBank under project number PRJNA494636 (accession numbers are presented in Fig. 3).

### Phylogenetic analysis of ST131 Group1 and Group2 complete sequence plasmids

Phylogenetic analyses of complete sequence plasmids (n = 40) were performed based on SNPs alignments generated by kSNP3.0^56^. Plasmid SNPs were retrieved from all IncF complete sequences. Maximum-likelihood trees were build using RAxML v8.2.12 with GTR CAT substitution model and 100 rapid bootstrap replicates^57^ and visualized using the online Interactive Tree Of Life (iTOL) v4^58^.

## Supplementary information

Supplementary file.
