# Supplementary material for: Meta-analysis of Pandemic Escherichia coli ST131 Plasmidome Proves Restricted Plasmid-clade Associations
Source: Sci Rep. 2020 Jan 8;10:36. doi: 10.1038/s41598-019-56763-7 (PMC6949217; doi:10.1038/s41598-019-56763-7)
Supplement: Supplementary file 1 — Supplementary file. [file 41598_2019_56763_MOESM1_ESM.pdf]

Supplementary Materials

Meta-analysis of Pandemic *Escherichia coli* ST131 Plasmidome Proves Restricted Plasmid-clade Associations

Kira Kondratyeva, Mali Salmon-Divon and Shiri Navon-Venezia*
Department of Molecular Biology, Faculty of Natural Sciences, Ariel University

*** Correspondence:** Prof. Shiri Navon-Venezia, shirinv@ariel.ac.il

Supplementary Table S1. Distribution of Group1 and Group2 replicons found in the SRA ST131 database (n = 880) among ST131 clades and CTX-M genes

Plasmid replicon	ST131 clade	CTX-M allele	N	Grouped
F1:A2:B20 (n = 245)	C1	27	77	Group1
		14	18	
		15, 27	3	Group1
		1	4	
		55	2	
		15	1	
		1, 9	1	
		no CTX-M	133	
	C2	15	1	
		no CTX-M	2	
	A	no CTX-M	2	
	UD	no CTX-M	1	
F2:A1:B- (n = 162)	C2	15	100	Group2
		14, 15	2	Group2
		19	1	
		3, 15, 52, 55	1	Group2
		no CTX-M	37	
	C1	14	1	
		no CTX-M	3	
	B	2	2	
		14	1	
		1	1	
		no CTX-M	3	
	UD	14	6	
		no CTX-M	4	

UD – Undefined

Supplementary Table S2. Group1 and Group2 plasmids from the NCBI Nucleotide database (last access 06.11.2019)

Plasmid	GenBank acc.	ST	Clade/ Virotype	Plasmid Group	FAB formula	CTX-M	Host	Source	country	year*
pEC732_2	CP015140	131	C1 / C	1	F1:A2:B20	27	human	UD	Thailand	2012
pEC542_1	CP018969	131	C1 / C	1	F1:A2:B20	27	human	UD	Vietnam	2011
pEC-81009	CP021180	131	C1 / C	1	F1:A2:B20	27	human	UTI	Emirates	2009
pH105	CP021871	131	C1 / C	1	F1:A2:B20	27	human	vaginal swab	Germany	<2017
p4_4.1	CP023827	131	C1 / C	1	F1:A2:B20	27	human	feces	Sweden	2009
pMO	MG886288	131	NR	1	F1:A2:B20	27	water	water	UD	<2018
p146-1	CP041573	131	C1 / C	1	F1:A2:B20	27	human	UD	USA	<2019
uk_P46212	CP013657	131	C2 / A	2	F2:A1:B-	15	human	UTI	UK	2005
p4_0.1	CP023850	131	C2 / C	2	F2:A1:B-	15	human	UTI	Sweden	2009
pEK499	EU935739	131	NR	2	F2:A1:B-	15	UD	UD	UK	<2008
pEC_L8	GU371928	131	NR	2	F2:A1:B-	15	human	UTI	Belgium	<2009
pEC_L46	GU371929	131	NR	2	F2:A1:B-	15	human	UTI	Belgium	<2009
pEC958	HG941719	131	C2 / A	2	F2:A1:B-	15	human	UTI	UK	2005
pEcoV282	KT988018	131	NR	2	F2:A1:B-	15	dog	cystitis	Germany	<2015
E. coli B36 plasmid 2	LR130546	131	C2 / A	2	F2:A1:B-	15	human	blood	Australia	<2018
E .coli VRES-hospital6495150 plasmid 1	LR595886	131	NR	2	F2:A1:B-	15	human	feces	UK	2014
E. coli TO124 plasmid 3	LS992182	131	C2 / A	2	F2:A1:B-	15	UD	UD	UD	<2018
E. coli TO148 plasmid 2	LS992191	131	C2 / A	2	F2:A1:B-	15	UD	UD	UD	<2018
NCTC 13441 plasmid	LT632321	131	C2 / A	2	F2:A1:B-	15	UD	UTI	UD	<2016
RCS102_p	LT985213	UD	NR	2	F2:A1:B-	15	UD	UD	UD	<2018
RCS22_p	LT985221	UD	NR	2	F2:A1:B-	15	UD	UD	UD	<2018
RCS59_p	LT985271	UD	NR	2	F2:A1:B-	15	UD	UD	UD	<2018

NR – not reported; was not able to be defined *in silico* due to absence of chromosome sequence.
UD – Undefined.
* For plasmids of which the year of isolation was not reported, we present the year (<) of GenBank submission.

Supplementary Table S3. ST131 clade-specific regions used for the identification clades *in silico*

E. coli ST131	Genbank acc.	Clade	Target*	Target size (bp)	Coordinates
CD306	CP013831	C0	self ST131	580	4609048..4609627
EC81009	CP021179	C1	self ST131	580	Complement(143280..143859)
			C1	337	Complement(4105735..4106071)
			C1-M27	232	Complement(102476 ..102707)
			C1 *aer*	140	1237294..1237433
JJ1886	CP006784	C2	self ST131	580	4621590..4622169
			C2 *ybbW*	194	564007..564200
EC958	HG941718	C2	self ST131	580	4641479..4642058
			C2 *ybbW*	194	566542..566735

*Target regions were extracted from the selected ST131 isolates using an alignment of clade-specific primers designed by Matsumura *et al.* (Rapid Identification of Different Escherichia coli Sequence Type 131 Clades. **Antimicrob. Agents Chemother**. 61, 2017)

Supplementary Figure S1. Association between *E. coli* ST131 plasmid and chromosomal features

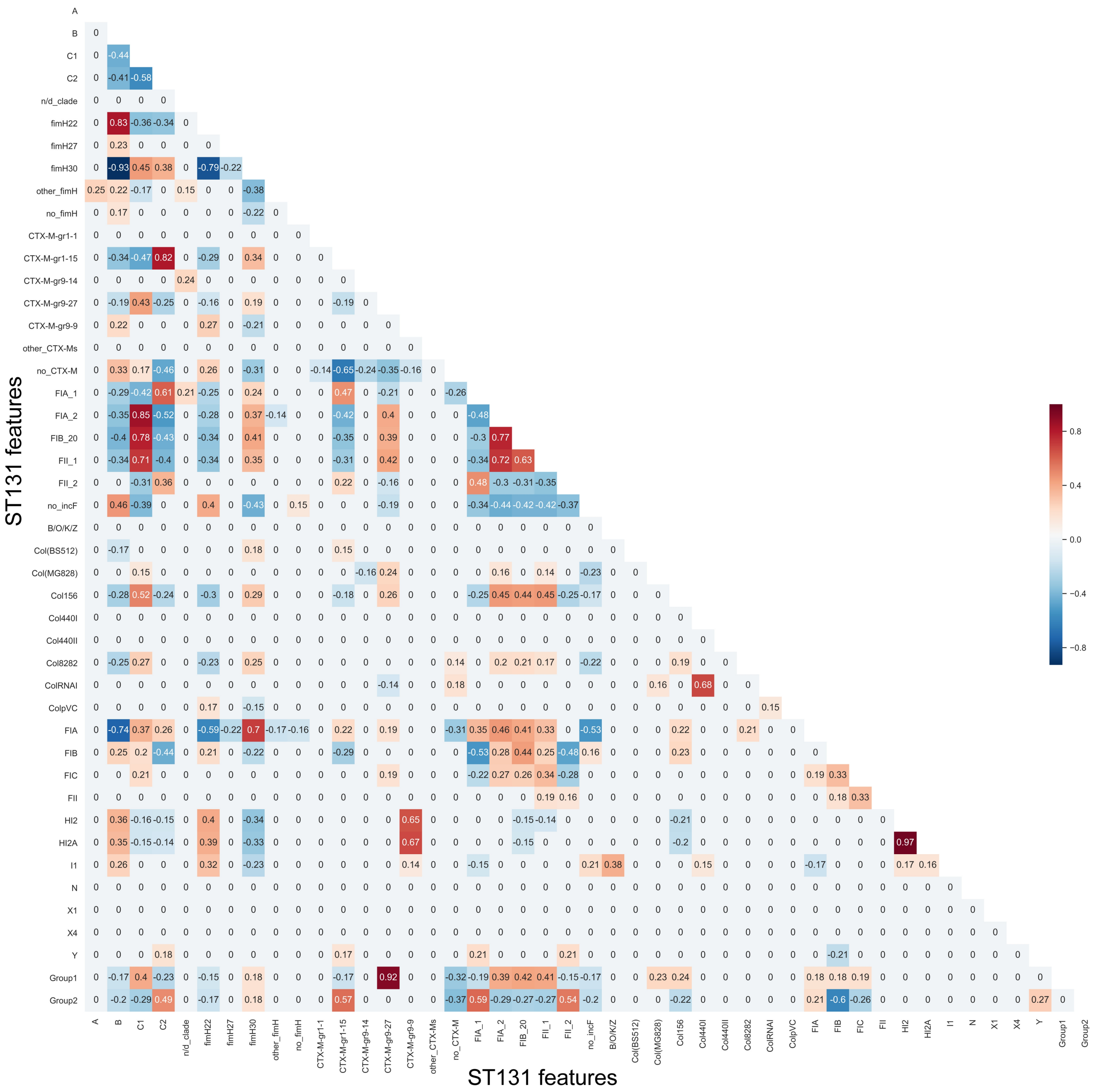

E. coli ST131 features paired associations. For each feature, a binary column (1- present/true, 0 - absent/false) was generated for all ST131 isolates (n = 880). phi coefficient was calculated for each pair of features. P-values were corrected in a P-value multiple test correction (step-down method using Bonferroni adjustments, alpha = 0.05). Coefficient values supported with corrected p-value <0.05 are shown in numbers, less supported results are masked with zeros.

n/d_clade – clade was not identified; no_fimH – fimH negative; no_CTX-M – CTX-M-gene negative; no_incF – IncF plasmid negative

other_fimH: 26 minor alleles, found in less than ten isolates

other_CTX-Ms: CTX-M-3, CTX-M-32, CTX-M-52, CTX-M-55, CTX-M-2, CTX-M-19, each found in less than ten isolates.

Supplementary Figure S2. PCA analysis of ST131 features

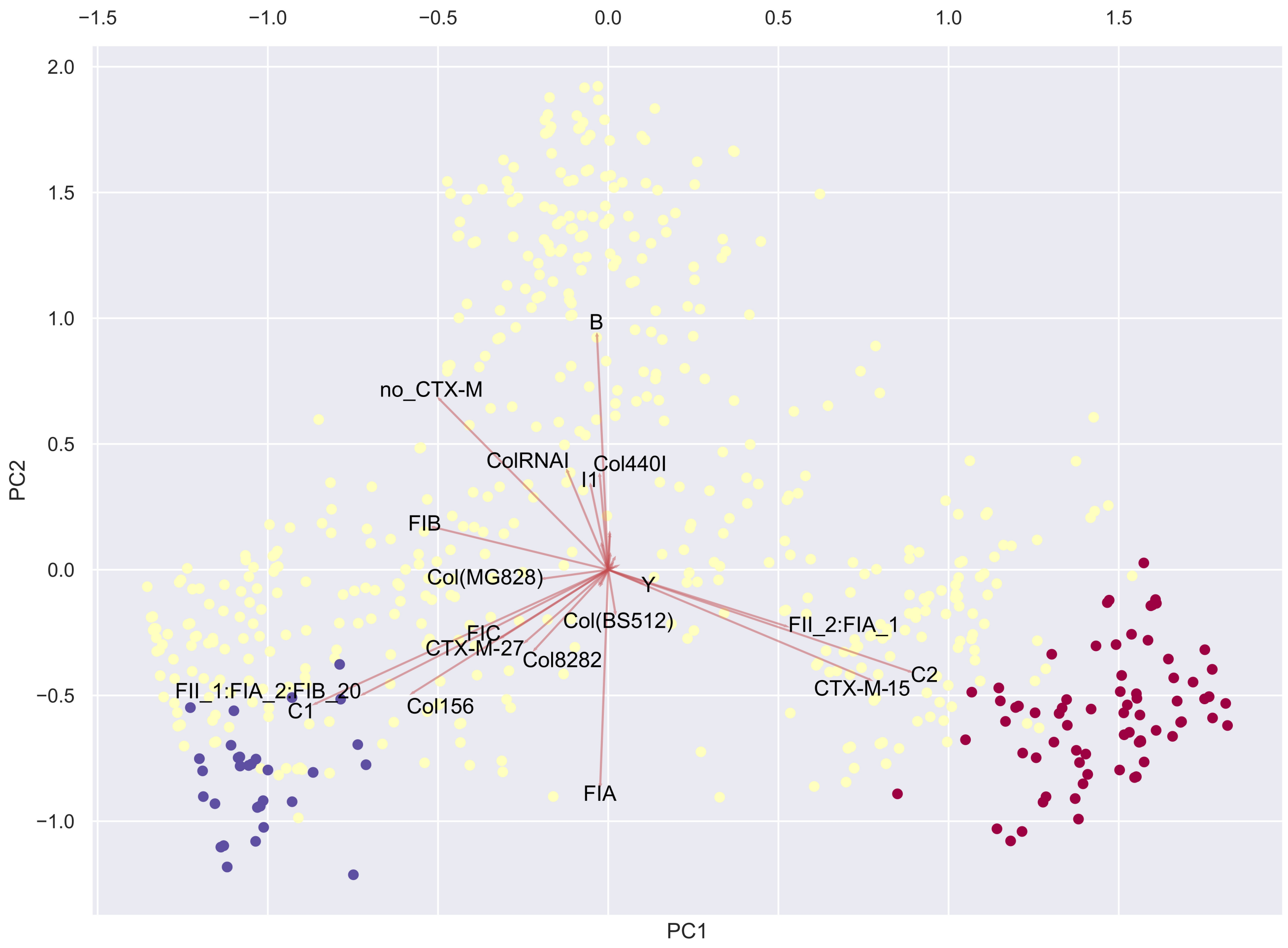

Principal-component analysis showing ST131 isolates (n = 880) presented as circles on the first two components. Isolates possibly carrying grouped plasmids are labeled with colors: Group1 – blue (n = 80), Group2 – red (n = 103).

For the analysis, we used a binary table of ST131 isolates in rows and genetic features in columns describing the presence or absence of each feature in each isolate. *fimH* alleles were removed from the data frame due to their definitive connection to the clades. Specific IncF replicons characteristic for Group1 and Group2 features were combined into single features. Features converted into loadings of different lengths are presented as red lines radiating from the center. The 19 features with the highest contribution to the isolates divergence are labeled with their names.

Supplementary Figure S3. Cross-coverage of complete sequences of Group1 and Group2 plasmids retrieved from the Nucleotide collection (nt/nr)

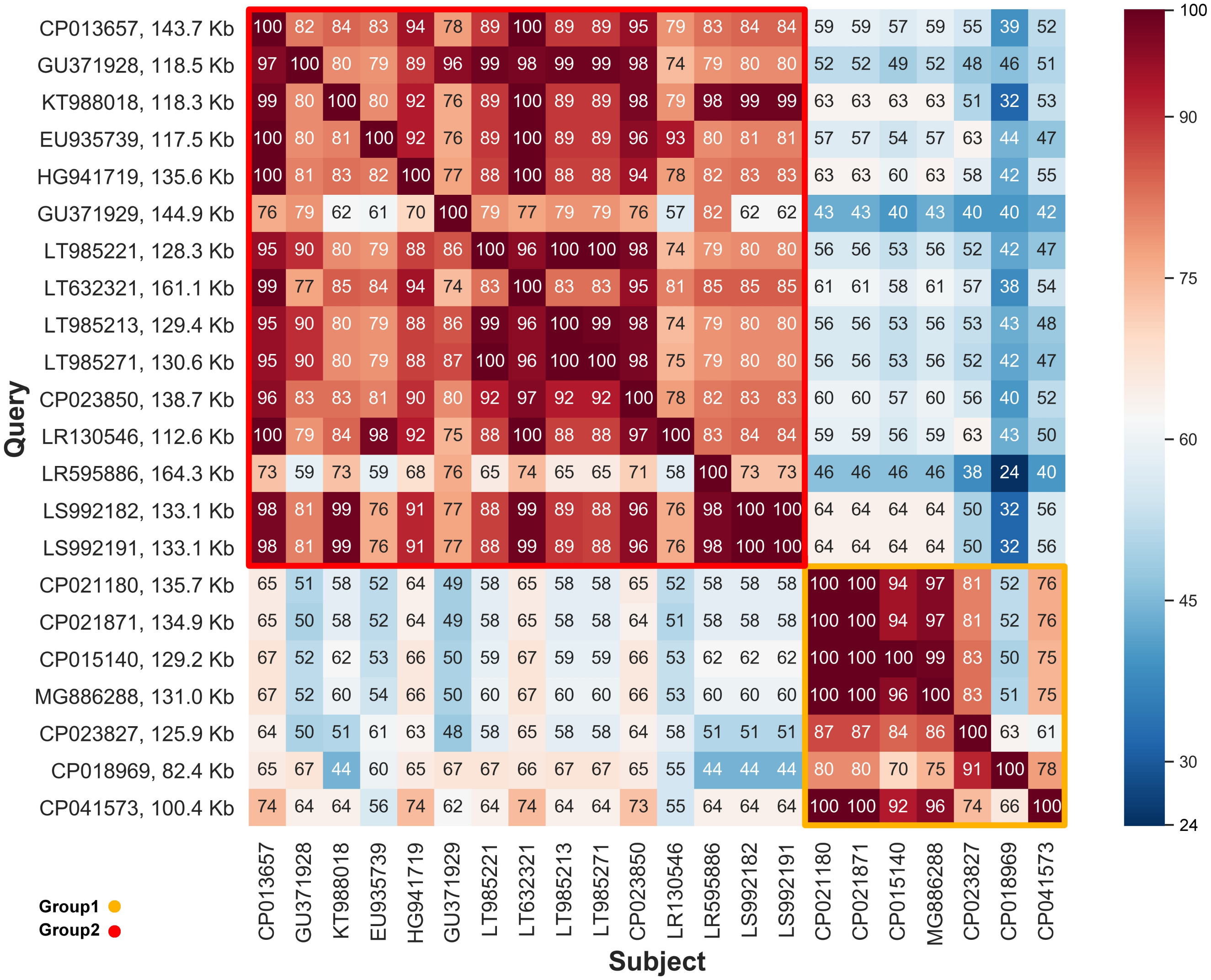

Group1 (n = 7) and Group2 (n = 15) plasmids were aligned all-to-all using BLASTn. The percent of the query coverage is presented in numbers. Accession numbers and plasmid sizes are presented on the y-axis.

Supplementary Figure S4. The coverage of Group1 and Group2 reference plasmids by grouped SRA *E. coli* ST131 isolates

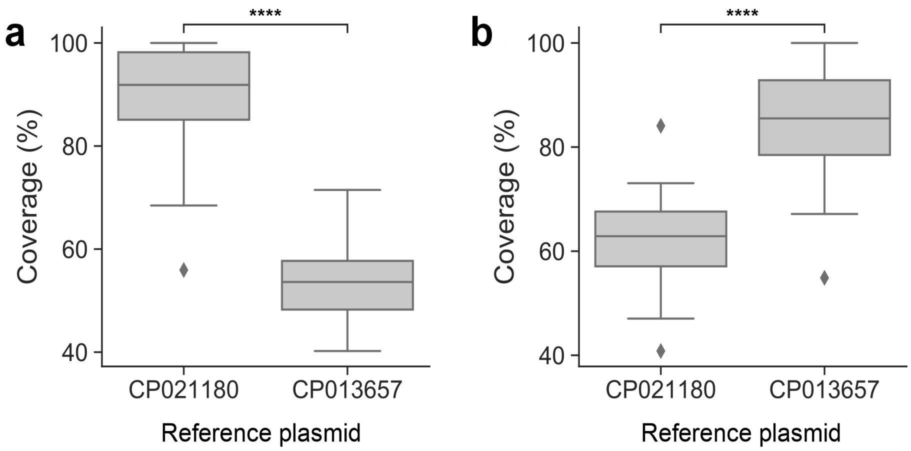

SRA WGS reads of isolates identified as ST131 Group1 (n = 80, panel A) and Group2 (n = 103, panel B) members were mapped to the Group1 (pEC-81109, Accession no. CP021180) and Group2 (uk_P46212, CP013657) reference plasmids. The box-and-whiskers diagrams present the median and quartiles of the coverage results. Comparison was performed using two-tailed Mann-Whitney test on non-normally distributed data. **** P-value < 0.0001

Supplementary Figure S5. Cross-coverage of Israeli ST131 plasmids

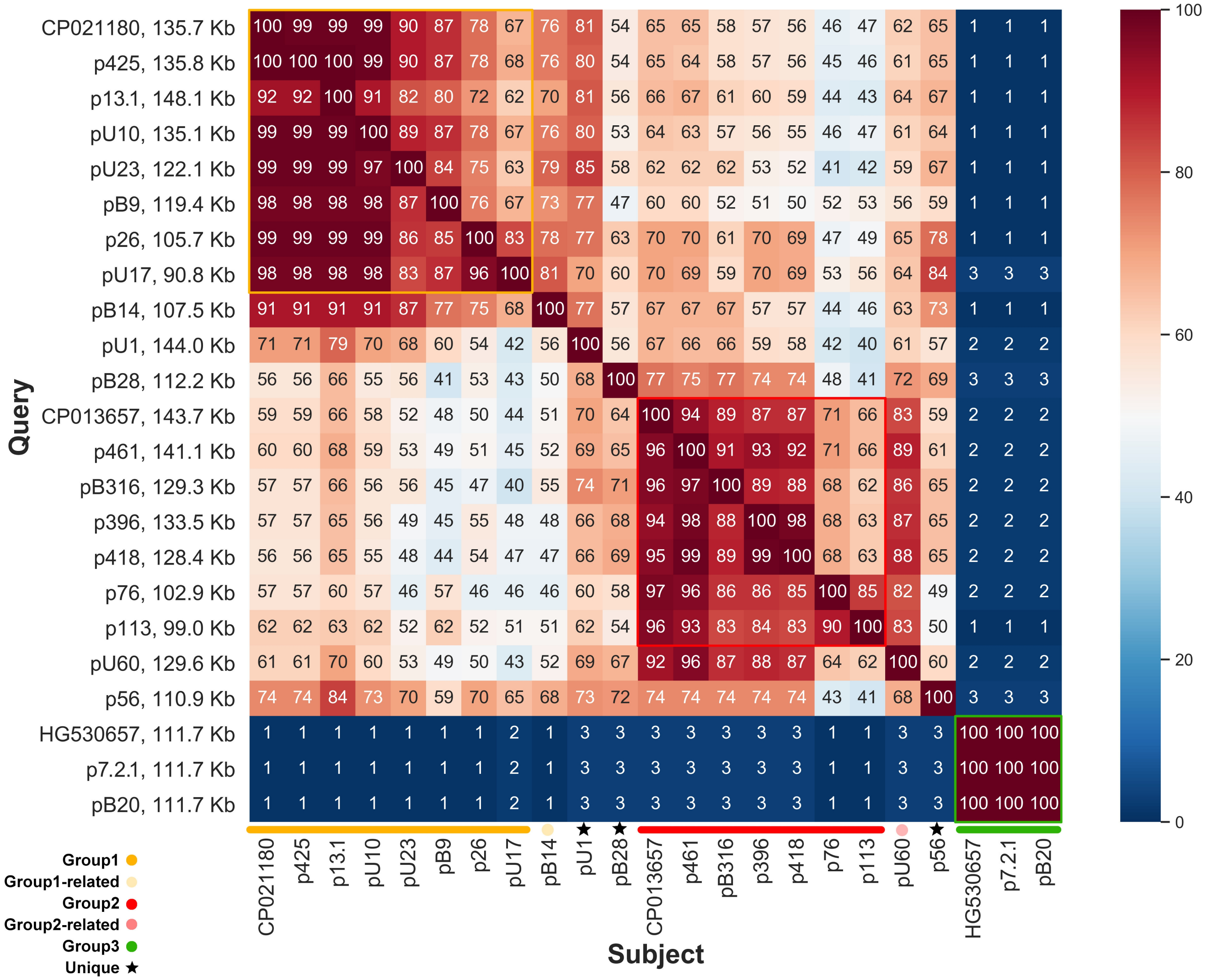

Israeli ST131 plasmids (n = 20) were grouped according to their replicon type and CTX-M allele and combined with respective GenBank reference plasmid. Group1 (n = 8), Group2 (n = 7), Group3 (n = 3) and ungrouped (n = 5) plasmids were aligned all-to-all using BLASTn, the percent of the query coverage is shown. Plasmids with slightly different replicon types were combined as group-related plasmids: pB14 (CTX-M-27 IncF[F1:A2:B-]) to Group1, and pU60 (CTX-M-15 IncF[F-:A1:B-]) to Group2. Other ungrouped plasmids were marked as unique. Plasmid names and sizes are presented on the y-axis.
